# Foreign connections and the difference they make: how migrant ties influence political interest and attitudes in Mexico

**DOI:** 10.1186/s40878-018-0098-y

**Published:** 2018-11-19

**Authors:** Lauren Duquette-Rury, Roger Waldinger, Nelson Lim

**Affiliations:** 1 University of California, Los Angeles, 264 Haines Hall, 375 Portola Plaza, Los Angeles, CA 90095-1551 USA; 2 Fels Institute of Government, University of Pennsylvania, 3814 Walnut Street, Philadelphia, PA 19104 USA

**Keywords:** Transnational migration, Political interest, Mexico, Social ties, Remittances

## Abstract

Beyond the economic and social effects of international migration researchers show regular exchanges between immigrants and stay-at-homes produce political spillovers in sending countries. As a broad body of literature demonstrates, most migrants maintain at least some form of contact with key connections back home, whether through long-distance communication, remittance sending, or in person visits. We investigate if exposure to international migration affects non-migrant citizens political interest, awareness, and attitudes about the efficacy of elections using longitudinal survey data from the Mexico 2006 Panel Study. We use a novel statistical approach combining Double Robust estimation technique with propensity score weighting. Our results suggest that Mexican non-migrant citizens exposed to international migration through social connections and remittances are more likely to be politically aware than those without. We also offer theoretical pathways to explain how ideational and material resources embedded in migrant social networks influences the political interest of stay-at-home citizens.

**Electronic supplementary material:**

The online version of this article (10.1186/s40878-018-0098-y) contains supplementary material, which is available to authorized users.

A network driven phenomenon, population movements across borders inherently and recurrently generate home country spillovers. While connections linking points of origin and destination cannot trigger migrations, once created they keep migration and information flowing: Cross-border connections enable informational exchanges between migrants and stay-at-homes about opportunities found abroad; support to newcomers; and the adoption of new forms of consumption, behaviors, and attitudes learned in the society of destination. For these reasons, international migration is a self-feeding, path dependent process, in which initiating causes reinforce feedbacks in both place of origin and place of destination. The transnational social networks that form around these connections between origin and destination are imbued with ideational and material resources affecting social, political, and economic life in origin countries.

Beyond the economic effects of international migration, which have been extensively examined, regular exchanges between migration and stay-at-homes produce social and political spillovers in sending countries. Most migrants maintain at least some form of contact with key connections back home, whether through long-distance communication or in person visits (Soehl & Waldinger, 2010). As the capacity for long-distance communication steadily grows – for reasons having to do with cost declines, the growing prevalence of communication technology in places of origin and technological changes making for more intimate contact (e.g. videocast) – and the costs of traveling also falls, these exchanges can yield the transmission of ideas, norms, expectations, skills and contacts acquired in the society of destination. Capitalizing on the interest in economic remittances, Peggy Levitt advanced the concept of “social” remittances to characterize the transmission of ideas, norms, values, and behaviors transmitted through migrant social networks (Levitt, 1998).

More recently, the concept of social remittances has given birth to the cognate idea of “political” remittances, in which the ongoing transnational exchanges between migrants and stay-at-homes serve as vehicles for “remitting” political experiences, ideas, values and expectations (Lacroix, Levitt, & Vari-Lavoisier, 2016; Piper, 2009). Unlike economic remittances – where the impact derives from the wage difference in sending and receiving country, for example – non-material remittances become channeled to the political arena only when the migration entails a move across institutionally distinctive polities. In this light, exposure to the disparate characteristics of the receiving polity, whether more democratic, peaceful, representative, participatory, accountable, or institutionally more predictable (or less), leads migrants to remit implicit and explicit political ideas, preferences, and behaviors related to their new, possibly “enriching” experiences back home to their compatriots (Batista & Vicente, 2011).

In this paper, we investigate if migrant transnational network ties affect non-migrant citizens’ political interest in and attitudes about Mexican elections. We focus on citizens’ interest in, following of, and attitudes about elections, all of which serve as an important precursor to formal political behaviors such as voting (Córdova & Hiskey, 2015; Rosenstone & Hansen, 1993; Verba, Schlozman, & Brady, 1995). While a growing body of research explores whether migration enhances (or stymies) political behaviors and attitudes in origin countries and has yet to reach consensus (Bravo, 2009; Careja & Emmenegger, 2012; Chauvet, Gubert, & Mesplé-Somps, 2016; Chauvet & Mercier, 2014; Córdova & Hiskey, 2015; Goodman & Hiskey, 2008; Meseguer, Lavezzolo, & Aparicio, 2016; Pérez-Armendáriz & Crow, 2010; Rother, 2009; Rüland, Kessler, & Rother, 2009), we add to this growing literature by focusing on the role of migrant transnational social ties and remittances in affecting non-migrants’ interest in and following of politics.

We assess the political consequences of international migration in Mexico, a democracy with substantial emigration, through an analysis of the Mexican 2006 Panel Study. The Mexican elections study is a high quality, nationally representative, longitudinal survey, fielded at two intervals prior to the national election and then a third time after votes were cast. Any presidential election provides a strategic opportunity for examining political attitudes, as the publicity and mobilization it generates awakens political interest that might otherwise lie dormant. That generalization especially applies to this particular election: the next to occur after the precedent breaking 2000 Mexican presidential election, in which the PRI – Mexico’s heretofore ruling party – was swept out of office for the first time since the Mexican Revolution. As a result, the 2006 election entailed intense political competition. Moreover, it provided the very first chance for Mexican citizens living abroad to vote in homeland elections. While a variety of factors kept emigrant participation low (Leal, Lee, & McCann, 2012), the election nonetheless stimulated interest among immigrants in the United States. Hence, in analyzing this survey we can gain the capacity to identify any migration-related effects at a time when political interest across the electorate is high. Additionally, the survey provides leverage on this question as, in addition to collecting standard political and demographic data, the 2006 survey also asked questions related to contact with migrant kin *and* receipt of remittances, making it possible to identify micro-level connections between emigrant relatives living abroad and non-migrants residing in Mexico and as well as the transfer of remittances from the former to the latter. Unfortunately, the 2012 survey did not include questions relating to immigration. The availability of data on *both* remittances and migrant social ties in the 2006 survey permits us to analyze the extent to which these cross-border connections yield independent effects on political interest or if more variation can be explained by considering the weight that these social and economic connections produce jointly.

We use a statistical approach called Doubly Robust (DR) estimation technique (Bang & Robins, 2005) that blends propensity score (PS) weighting and familiar linear regression models. This approach helps to mitigate the selection effects often associated with emigration and political outcomes since the DR technique is a rigorous approach that controls for observed confounders using observational data. As the name suggests, the DR technique is superior to either the PS analysis or regression models alone because it yields results that remain consistent even if either of the estimations are misspecified.

We find compelling evidence that Mexican citizens with migrant social networks ties are more likely to report being interested in politics, talking about politics, and have more critical opinions of Mexican elections than Mexican voters without migrant ties. However, familial social ties and receiving monetary remittances do not impart the same political effects across interest in, awareness of, and attitudes towards Mexican elections. First, Mexican voters with one or more close relatives in the U.S. but who do not report receiving migrant remittances are more likely to report talking about politics than individuals without family ties to migration. Second, receipt of remittances, though it does not appear to affect political talk, does affect the likelihood that respondents report being interested in politics: those non-migrants with relatives in the U.S. *and* who also receive remittances are more likely to be interested in politics and be critical of Mexican elections than either individuals with *only* a family member abroad or absence of any migrant network ties, all other things equal. In general, we find that individual exposure to international migration drives political interest in and attitudes about of Mexican elections, although social ties and remittances have independent and joint effects.

We structure the paper as follow. First, we theorize how migrant cross-border connections affect political awareness of stay-at-home citizens through several potential pathways. Next, we discuss the research design and methodological approach followed by a presentation of the results and a discussion of their significance. Finally, we examine how well our findings on international migration and political awareness in the Mexican context may travel to other countries with extensive out-migration.

## Migrant cross-border connections and political remittances in Mexico

The U.S.—Mexican migration corridor is the most frequently crossed border in the world. As of 2012, 11.4 million immigrants born in Mexico reside in the United States accounting for two thirds of the U.S. Hispanic population (Gonzalez-Barrera & Lopez, 2013). As a large body of research shows, in addition to retaining cultural pride for the homeland and maintaining religious practices across borders (Smith, 2006), most migrants also stay connected to Mexico either through return visits, phone calls and video conferencing or sending monetary remittances (Waldinger, Soehl, & Lim, 2012). As of 2016, remittances to Mexico hit a record $27 billion surpassing oil revenues for the first time (Ratha, 2016).

More recently, researchers have begun to consider the political consequences of international migration for countries of origin. Researchers are assessing how absentee voting affects election outcomes (Leal et al., 2012; McCann, Cornelius, & Leal, 2009; Nyblade & O’Mahony, 2014; O’Mahony, 2013), how migrant remittances affect governance and public goods provision (Adida & Girod, 2010; Burgess, 2012; Duquette-Rury, 2014; Duquette-Rury, 2016; Pfutze, 2012); and how migrant absence and return affect political engagement and attitudes about democracy and public policy (Bravo, 2009; Chauvet & Mercier, 2014; Chauvet et al., 2016; Córdova & Hiskey, 2015; Dionne, Inman, & Montinola, 2014; Duquette-Rury & Chen, 2018; Goodman & Hiskey, 2008; Meseguer et al., 2016; Pérez-Armendáriz, 2014; Pérez-Armendáriz & Crow, 2010). Since researchers have been largely remiss in characterizing the political features of international migration for sending countries, this growing body of research is a welcome addition to the inter-disciplinary literature on the causes and consequences of international migration. Still, there is a great deal more we need to understand about how international migration influences the political landscape in migrant origin countries.

We build on this existing research and identify the extent to which non-migrant citizens’ social networks ties and remittance transfers translate into political interest during an election campaign. Since, as Verba et al. (1995) argue, political interest is often a prerequisite of political participation, the extent to which citizens are politically interested tells us something about how likely they may be to participate in formal and non-electoral forms of politics (Córdova & Hiskey, 2015). Those indirect channels of influence linking migrants abroad to politics at home, via their non-migrant relatives, are likely to comprise the key vector of migrant political influence, at least in Mexico, as absentee voting of Mexican nationals in the U.S. has been shown to do very little to directly affect elections (Leal et al., 2012; McCann et al., 2009). Thus, we advance the literature by showing how migrant remittances and social networks modify the Mexican electorates’ political interest and awareness during presidential elections.

The bulk of the comparative scholarship in the U.S. and beyond explains political interest and engagement using the standard socioeconomic model – education, income and occupation. Since interest and attitudes are the property of individual preferences and psychological orientations, much of the literature concentrates on the predictive capacity of different socioeconomic characteristics. However, other studies convincingly show how individual political orientations are also affected by information sharing, recruitment, and mobilization occurring through inter-personal social connections among acquaintances, friends and relatives, and institutional features of the political system that both constrain and encourage interest and engagement (Holzner, 2007; Rosenstone & Hansen, 1993). In this article, we analyze how interested citizens are in politics, how important politics is in their daily lives, and the degree to which they believe in the efficacy of Mexican elections. While we cannot evaluate every measurable facet of political engagement in this study, for example, political behaviors, we suspect, following the seminal research of Rosenstone and Hansen (1993) and Verba et al. (1995), that non-migrant political interest informs some understanding of political behaviors and personal political efficacy.

## Theoretical expectations

As Baker (2006) finds in his analysis of regionalized voting behavior in Mexican national politics, voters do not make political decisions in a social vacuum. Rather, the Mexican electorate makes political decisions amidst ongoing interpersonal interactions and exchanges with political discussion partners. Baker contends that the voting public discusses politics and “openly deliberate [s] over their choices with family and friends, accepting advice and new information from others while at times attempting themselves to persuade...citizens are embedded in social networks that sustain politically relevant interpersonal exchanges” (Baker, 2006, p. 6). Those networks can span territorial boundaries because in opting for life in a new country and leaping over the borders separating home and host societies, international migrants paradoxically knit those societies together (Mouw, Chavez, Edelblute, & Verdery, 2014).

### Cross-border communication and political learning

Cross-border exchanges are not universal, but are nonetheless prevalent, as only a minority of migrants completely severs the ties to close associates still living in the country of origin. Although over time, family networks tend to shift the center of gravity from sending to receiving country, that process is long, uncertain, and rarely complete. Inertia exercises significant influence on the location of kinship ties: older parents are less likely to leave; in turn, their continuing home country residence is a constraint on the movement of others, as demands for parental caring can keep adult children in place. Those persisting ties motivate continuing contacts; as noted in a recent study of migrants in Spain, frequent contact is more prevalent among those with immediate kin still in the home country as opposed to those whose closest family ties had undergone emigration (Park & Waldinger, 2017).

These ongoing, long-distance, conversations provide one channel for the flow of political information. As shown by Pérez-Armendáriz (2014) in a study of cross-border conversation among Mexican immigrants in the United States and their relatives at home, those exchanges principally revolved around practical matters, related to family well-being, health, everyday life, and future plans. Nonetheless, in-depth analysis of these same conversations showed that these discussions contained political content, as the emigrants conveyed both information about politically relevant experiences in the United States as well as opinions regarding the political implications for Mexico.

Other forms of cross-border connectedness provide a more proximal basis for the acquisition and transmission of political information. Though visiting is more occasional than communication, home country travel is widespread; those in-person visits will yield opportunities for the transmission and acquisition of political information that can only be gleaned in situ, as when a visit coinciding with a homeland political campaign brings the migrant face-to-face with the politics that she had left behind. Moreover, migration itself may trigger homeland responses that directly transmit political signals. Thus since long-term, large-scale migrations frequently yield return visits that are recurrent and patterned, as in the annual pilgrimages made by countless Mexican migrants for a 1 week celebration of their hometown’s patron saint (Massey, Alarcón, Durand, & González, 1987, pp. 143–145), they can also lay the basis for institutionalized contact with homeland political leaders, who make their presence known to the otherwise absent sons and daughters (Fitzgerald, 2008). Last, the migratory circuit itself may yield a strong sense of home community membership, as exemplified by the growing number of hometown associations. Though these organizations are locally focused, oriented towards philanthropy, they necessarily connect migrants and their hometown networks with politics (Duquette-Rury, 2016).

Thus, the persistence of cross-border ties yields political inputs, providing migrants with multiple opportunities to stay abreast of developments at home, years of physical separation notwithstanding. Those connections also provide the channel for political outputs, via communication of the lessons learned as a result of the experience of movement to a more democratic polity with better functioning institutions. International migrants all begin as foreigners, and therefore spend some significant portion (possibly all) of their lives in the destination country outside the polity. Nonetheless presence does yield basic personhood rights: foreigners have the capacity to engage in a broad variety of non-electoral, political and civic activities; as they often do so (though at highly variable rates), these modes of participation can provide instruction in democratic processes and also increase a sense of individual political efficacy. Indeed, as found by Pérez-Armendáriz, Mexican immigrants communicating with their relatives abroad shared “their understandings of and experiences with public and political life in the USA, including norms, values, and practices” (2014, p. 75).

Fostering the development of those understandings are the efforts maintained by numerous organizations – unions, schools, and civic associations – to reach out to or mobilize immigrant community members regardless of legal status in the destination country (Leal, 2002). Since foreigners are not segregated into migrant-only communities, but rather live alongside (and often with) citizens, they gain exposure to the political messages directed at otherwise similar neighbors who, however, possess the right to vote. As demonstrated by the immigrant rights marches that swept U.S. cities in 2006, those messages can be directed at a non-citizen population with great effect (Zepeda-Millán, 2017).

Second, citizenship acquisition and the political opportunities it creates can yield even greater effects. The very process of acquiring citizenship entails greater attention to the destination polity and its characteristics (Waldinger & Duquette-Rury, 2016). New citizens obtain the right to vote, which, when exercised, entails a significant uptick in political activity, and probably attention, relative to the prior years of exclusion from the polity. Insofar as citizenship acquisition is one component of a broader process of assimilation in which immigrants’ exposure to the citizenry progressively grows, so too will their connection to networks of more politically engaged persons from whom more political information may flow.

Consequently, we hypothesize that:

- **H1:** In Mexico, persons with ties to relatives living outside the country will exhibit higher levels of interest in politics as compared to those with no ongoing cross-border social ties.
- **H2:** In Mexico, persons with ties to relatives living outside the country will talk about politics more frequently with discussion partners as compared to those with no ongoing cross-border social ties.
- **H3:** In Mexico, persons with ties to relatives living outside the country will exhibit more critical views of Mexican politics as compared to those with no ongoing cross-border ties.

As noted above, the unbundling of cross-border ties is often a highly protracted process, with the result that migrants long retain connections and commitments to the people and places left behind. Those connections provide the channels for the circulation of information, ideas, and opinions going back and forth between place of origination and destination. Yet one still has to ask why migrants retain an interest in home country political matters and why stay-at-homes might heed the preferences of their relatives who have opted for life in another country.

The likely answer lies in an insight conveyed by the “new economics of labor migration”: namely, that the very decision to leave home was embedded in family-level processes, which subsequently exercise long-term influence (Stark & Bloom, 1985). Cross-border ties frequently advance the ends of both migrants and stay-at-homes since in developing societies, emigration is often undertaken without the goal of immigration: rather, relocating to a developed society takes place so that emigrants can gain the access to the resources that can only be found there. In turn, those gains get channeled back home in order to stabilize, secure, and improve the options of the kin network remaining in place. The stay-at-homes are not just receiving help, but also providing it, whether caring for the children or elderly parents left behind, attending to the house that the migrant has built with her remittances or the property that he owns (or hopes to inherit), providing assistance when trouble strikes in the host country or some home country document is needed to stabilize the host country situation. These interdependencies give the migrants a stake in political developments on the ground back home, while similarly disposing the stay-at-homes, receiving help from the migrants, to respond to the latter’s preferences.

The intertwined survival strategies of both migrants *and* stay-at-homes might explain both why migrants might be motivated to communicate political lessons learned abroad and why stay-at-homes would be inclined to listen to those messages. If so, the set of persons responding most intently to messages transmitted from the country of immigration will only comprise a subset of all persons with ties to migrants abroad, as with settlement and the shifting of key kinship ties from home to host societies cross-border interdependency drops.

The sending of remittances lies at the core of those interdependencies. Though cross-border communication – whether by phone, social media, or email – is more common than the material-demanding sending of remittances, remittances comprise a prevalent migrant social practice of huge economic significance. Remittances are typically transmitted electronically and, strictly speaking, convey money, not talk. Nonetheless, the sending of remittances, as Lacroix (2015) has contended, inherently involves a communicative act and one that is embedded in the ongoing, complex negotiations that keep long-distance, cross-border contacts alive. The sending of remittances also comprises but one piece in a set of connected interchanges, as remittances are often triggered by requests for help from recipients, whereas senders are likely to contact receivers to verify that the funds have arrived and to inquire into their use. Moreover, while remittance sending may be stimulated by material concerns, the multi-faceted nature of remittances – involving a broad range of scripts, as contended by Carling (2014) – as well as the likely ambiguities as to how these changes are to be understood at both sides of the chain, suggest that communications concerning the flow of money are unlikely to focus on that matter alone. And as noted above, even though those communications are likely to pivot around practical matters, political issues are often present.

Distance allows the migrants to exercise influence, but not control, which is why the way in which remittances are spent often emerges as a source of conflict in the cross-border relationship: in particular, migrants prefer that remittances be used for savings or investment, whereas recipients show a preference for consumption. Paradoxically, the response to this dilemma takes the form of the frequent sending of relatively small sums, a strategy that imposes significant material costs (in the form of a transaction fee), but may also be one that provides the migrant with greater control (Yang, 2011). Yet, for our concerns, this same strategy suggests that remittance senders and receivers will be in more regular contact than those stay-at-homes who are managing without assistance from relatives abroad. Hence, the relationship between remittance senders and receivers may be one that is particularly conducive to the transmission of political messages from societies of immigration to societies of emigration. On the other hand, just as recipients tend to have the last word over how remittances are spent, so too can they decide how to respond to political signals sent from the immigrants, possibly concluding that the monies coming from abroad let them buy the resources that governments might otherwise provide, making political detachment rather than interest the more rational response.

Consequently, we hypothesize that:

- **H4:** In Mexico, the combination of cross-border ties *and* remittance receipt will heighten political interest: persons with relatives living in the United States *and* who also receive remittance will exhibit greater interest in Mexican politics, as compared to those who have relatives in the United States but do not receive remittances as well as those with no ongoing cross-border ties.
- **H5:** In Mexico, the combination of cross-border kinship ties and remittance receipt will increase political talk: persons belonging to cross-border kinship networks *and* who also receive remittance will talk with discussion partners more frequently about Mexican politics, as compared to those who only belong to cross-border kinship networks but do not receive remittances as well as those with no ongoing cross-border ties.

## Research design and methodology

We use Doubly Robust (DR) estimation technique in all our specifications (Bang & Robins, 2005). DR estimation is a technique to estimate treatment effects in observational studies across a treated and non-treated control group. Since the treatment effect, in this case exposure to international migration through migrant social ties and remittances, is not randomly assigned into the two groups, it may be the case that the two groups are different in pretreatment characteristics that results in selection bias. The DR method combines both propensity score matching (PS) with regression analysis to alleviate common concerns about selection effects when using observational data.

Using both the propensity score weights with statistical regression models we are able to approximate, holding all other observed factors constant, what the political interest of respondents without migration exposure (control group) would be if they would have had ties to migrants abroad and/or received remittances (treatment). The DR approach thus allows us to create two groups of respondents that are most similar but for their exposure to migration to isolate the potential effects of migrant social ties and remittances on political interest, talk, and attitudes of non-migrant citizens in Mexico. Additionally, the DR estimation controls for a wide range of potentially confounding variables that relate to both migration and the political outcomes of interest and the resulting estimates are robust against failures to meet assumptions about propensity score models and regression models. This approach gives us greater confidence that the causal inferences we draw from the Mexican elections data are more likely attributed to migration exposure than individual level characteristics associated with who migrates.

There are several steps in the DR estimation technique. The first step is to compute a propensity score for each respondent in the control group. A respondent’s propensity score is her probability of being in the treatment group (having a US social tie or receives remittances), conditional on the observable characteristics. Rosenbaum and Rubin (1983) show that we can remove confounding influence of the observable characteristics by comparing outcomes of observations in the treatment and control groups with similar propensity scores (Rosenbaum & Rubin, 1983). We estimate propensity scores for various comparisons using an approach proposed by McCaffrey, Ridgeway, and Morral called the generalized boosted model (GBM) (McCaffre, Ridgeway, & Morral, 2004). The GBM is a flexible, nonparametric estimation technique, based on regression trees, that captures the relationship between the respondents’ characteristics and the treatment indicator.^1^ Because of its flexibility and fewer assumptions needed compared to linear logistic regression models, the GBM outperforms alternative models in comparison analysis (Westreich, Lessler, & Funk, 2010).

Once the GBM estimates the propensity scores, they can be used to weight respondents in the control/comparison group to match the distribution of observed characteristics of the treatment group, or

$$ f\left(x| treatment\ group\right)=w(x)f\left(x| control\ group\right), $$

where *x* is the vector of observed characteristics. McCaffre et al. (2004) show that, solving for *w*(*x*) and applying Bayes theorem produces the following results:

$$ w(x)=\left[\frac{f\left(t=1\right)}{f\left(t=0\right)}\right]\left[\frac{f\left(t=1|x\right)}{1-f\left(t=1|x\right)}\right] $$

The above equation suggests that in order to remove any difference in observed characteristics between the two groups, respondents in the control group should receive a weight equal to

$$ \frac{\mathit{\Pr}\left( treatment|x\right)}{1-\mathit{\Pr}\left( treatment|x\right)} $$

which are the odds of being a Mexican citizen with cross-border connections given the observed characteristics. Since we are using the survey sample, we multiply these propensity score weights with survey weights (Dugoff, Schuler, & Stuart, 2014).

The effectiveness of using these propensity score weights to balance the treatment and control group is apparent in Table 1. The distributions of observed characteristics between observed “Migrant Respondents” and “weighted non-Migrant Respondents” are more similar than the distributions between observed “Migrant Respondents” and observed “non-Migrant Respondents.”

**Table 1:** Distribution of Selected Characteristics by Migration Treatment and Control Groups

	Non-Migrant Families	Migrant Families	Weighted Non-Migrant Families
Gender			
Female	54%	47%	48%
Male	46%	53%	52%
Race			
White	19%	19%	20%
Light brown	48%	51%	50%
Dark brown	32%	29%	29%
Other	0%	0%	0%
No response	0%	0%	0%
Education			
No schooling	6%	6%	6%
Incomplete primary	17%	16%	14%
Complete primary	15%	16%	17%
Incomplete middle school/technical school	4%	2%	5%
Complete middle school/technical school	19%	22%	19%
Incomplete high school	7%	5%	7%
Complete high school	12%	13%	13%
Incomplete college	7%	6%	7%
Complete college or more	13%	13%	12%
Income			
0 a 1,299	15%	13%	12%
1,300 a 1,999	11%	12%	11%
2,000 a 2,599	11%	8%	8%
2,600 a 3,999	14%	14%	16%
4,000 a 5,199	12%	13%	13%
5,200 a 6,499	9%	11%	10%
6,500 a 7,899	6%	7%	8%
7,900 a 9,199	6%	6%	6%
9,200 a 10,499	5%	4%	5%
10,500 or more	10%	12%	11%
Region			
North	13%	21%	24%
Center	16%	29%	32%
Metro	29%	10%	7%
South	43%	40%	37%
Age			
17-30	31%	35%	35%
31-45	34%	35%	32%
46+	35%	31%	32%
City Type			
Urban	67%	71%	69%
Rural	26%	22%	21%
Mixed	7%	7%	10%
Marital Status			
Single	23%	26%	26%
Married	56%	58%	56%
Other	21%	17%	18%
Religiosity			
More than once a week	47%	47%	47%
Once a week	17%	18%	17%
Once a month	29%	31%	30%
Only on special occasions	6%	4%	5%
Never	1%	1%	1%

Source: Lawson et al. (2007). Authors' calculations using doubly robust estimation with propensity score weighting in R.

Although the weighted comparison does attempt to control for the observed characteristics, we take an additional step and perform a DR analysis with a weighted generalized logistic regression (GLM) controlling again for observed characteristics. As we stated, the DR methods are superior to either the propensity score weighted comparison or the parametric regression alone because the results remain consistent even if either the propensity score model or the regression is misspecified. In each specification, the estimator computes the average difference between each treatment respondent’s outcome and what the GLM predicts would be the respondent’s outcome had they been in the control group.

## Data and measurement

We assess the role of international migration on political interest, political talk, and attitudes in Mexico using data from the Mexico 2006 Panel Study (Lawson et al., 2007). The Mexico Panel Study is a national survey instrument fielded between 2005 and 2006 by Reforma newspaper’s Polling and Research Team. Team members conducted in-person interviews with selected Mexican voters at their residences before and after the presidential elections held July 2nd 2006. Respondents selected for the first panel wave (response rate was 34%) were re-contacted once again prior to the national election and once after the election for a total of three panel waves; the re-contact rates for the second and third panel waves were 74 and 67%, respectively. In addition to a national survey sample (*N* = 1600), two additional oversamples were collected including one for Mexico City (*N* = 500) and one for villages in rural areas in the states of Chiapas, Jalisco, and Oaxaca (*N* = 300). In total, 2400 interviews were conducted for the first wave and 1776 and 1594 respondents were successfully re-interviewed in the second and third waves, respectively.^2^ The multiple panel waves before and after the national election gives us an opportunity to assess how migration exposure affected political awareness and attitudes over the course of the electoral campaign.

### Measuring international migration

The survey asked participating respondents questions related to candidates, political parties, policy preferences, mass media, and political engagement as well as a few questions about international migration relevant to our study. In the first wave, participants were asked whether they had a close relative living in the United States, while in the second wave, re-contacted respondents were asked whether they or anyone in the household received money from someone living in the United States. These two questions form the bases of two related but theoretically distinct “treatment effects” of international migration exposure we evaluate in the analyses.^3^

In separate estimations, we study both independent and joint effects of having a relative living in the US and receiving remittances during the presidential election cycle. While other studies have argued that having a close relative in the U.S. and receiving remittances is correlated and thus including both measures would lead to multicollinearity and inflated standard errors in OLS model estimates (Bravo, 2009), we believe that having a relative in the U.S. *and* receiving remittances, as opposed to only having a relative in the U.S., might convey different kinds of political information affecting non-migrant political interest. In our sample, it is the case that receiving remittances and having a U.S. relative is correlated,^4^ as individuals receiving remittances must know someone in the U.S. in order to receive contributions from migrants, but there is no evidence of multicollinearity. We proceed by studying first, if international migration affects the political interest of non-migrants in any capacity and second, if there is something systematically different in the political awareness of those respondents that also receive material resources from kin abroad in the U.S. Since we use the doubly robust estimation technique in conjunction with propensity score matching to avoid selection bias in our estimations, inflated standard errors arising from multicollinearity is less of a factor in our estimations and we are able to evaluate the independent and joint effects of having a relative in the U.S. and receiving remittances in the logistic regression. In the present sample, the majority of respondents do not receive remittances from the U.S. (87%), but many do have a close relative living in the U.S. (46%). Of those respondents that report having a U.S. relative, 20% also receive remittances while 80% do not. This provides additional support for analyzing the effects of social ties and material transfers separately.

In addition to controlling for standard socioeconomic predictors of political interest including income, education, and race we also account for other individual and contextual covariates, which have been shown to affect political behavior.^5^ First, as Baker (2006) and Klesner (1995) show, Mexico has deep regionalized political cleavages related to wealth disparities, urban-rural cleavages, political discussion partners and religion that affect voting behavior (Baker, 2006; Klesner, 1995). We include dummy variables for Mexican region (north, center, metro, south), omitting one region in the analysis. The region variables also provide a control for areas with higher level of Mexican emigration as traditional Mexican sending states are concentrated in the central-western part of the country. Second, we include categorical measures of marital status, age, gender and urban residency to account for the effects of social connectedness, urbanization and other demographic characteristics on political interest. Finally, we capture a measure of religiosity in the form of frequency of church attendance, which is often a significant predictor of political interest.

### Political interest, political talk and the efficacy of Mexican elections

The Mexico 2006 Panel Study provides two distinct measures of political awareness, which we use in our analysis. First, we include a measure of political interest based on the question: How much interest do you have in politics? (a lot some, a little, or none). We use a dichotomous measure of political interest which takes the value of 1 if the respondent reports having any interest in politics and 0, otherwise. Second, we evaluate the frequency with which respondents’ report talking about politics with other people based on the following question: How often do you talk about politics with other people? (daily, a few days a week, a few days a month, rarely, never). Again, we dichotomize the outcome variable and code the value as 1 if respondents report talking about politics at all, and 0 otherwise. We use binary dependent variables in the statistical analysis for ease of interpretation using the DR approach, which is challenging to interpret using categorical dependent variables. However, we also assess the full categories for political interest and political talk as continuous variables and discuss these results in the next section.^6^ Finally, we evaluate the role of international migration on opinions of Mexican elections. The survey asks respondents if they agree or disagree that elections in Mexico are free and fair. The dependent variable is a dichotomous variable that takes the value of 1 if respondents agree and 0 if they disagree.

## Results

As described in the data section, the DR estimator first estimates the propensity scores by GBM and then fits weighted general linear models using the propensity score weights as observation weights. We also include survey weights in both the PS estimations and the weighted regression models. The DR estimator computes the average difference between each treatment case outcome (migration ties and remittances) and what the GLM predicts *would be* the respondent’s outcome had the respondent been in the control group. We report the DR estimations for political interest, political talk, and efficacy of Mexican elections for each wave and the effects of migration exposure in Table 2.^7^

**Table 2:** Doubly Robust Estimations of Migration Treatments on Political Interest, Talk and Efficacy of Mexican Elections, Panel Waves 1, 2 & 3

	**Wave 1**				**Wave 2**				**Wave 3**			
	Unweighted Average Outcome		Weighted Average Outcome for Treated *if in* Control		Unweighted Average Outcome		Weighted Average Outcome for Treated *if in* Control		Unweighted Average Outcome		Weighted Average Outcome for Treated *if in* Control	
	Treatment Group	Control Group		DRϕ	Treatment Group	Control Group		DRϕ	Treatment Group	Control Group		DRϕ
Interest in Politics												
Treatment 1	72.65	69.22	78.46	–5.81	82.95	77.53	83.02	–0.077	84.74	79.64	84.79	–0.058
Treatment 2	66.67	71.03	74.07	–7.41^.^	84.7	79.76	78.14	6.52^.^	83.45	81.79	75.66	7.79*
Talk about Politics												
Treatment 1	43.01	36.67	38.61	4.41**	55.36	46.13	56.18	–0.82	49.82	41.36	44.86	4.96*
Treatment 2	32.02	40.86	37.07	–5.05	48.88	51.25	46.52	2.36	36.55	47.01	42.92	–6.36
Treated Sample Size	847	Effective Sample Size			208							
Elections are Free and Fair												
Treatment 1	−	−	−	−	−	−	−	−	76.54	71.14	79.35	–2.81
Treatment 2	−	−	−	−	−	−	−	−	75.91	74	83.99	–8.08*
Treated Sample Size	115	Effective Sample Size			497							

Source: Lawson et al. (2007). Authors' calculations using doubly robust estimation with propensity score weighting in R. ϕ Doubly robust estimator is the unweighted treatment group average minus the weighted control group average. Note: Effective sample size is after weighting. Signif. codes: *p* < 0.10 *p < 0.05 **p < 0.01 ***p < 0.001

In Table 2, we report the unweighted average outcomes for both the treatment and control groups, the weighted average outcome for the treatment group *if they had been* in the control group and the DR estimator, which is the difference between the unweighted average outcome for the treatment group and the weighted average outcome for the treatment group if they were in the control group. In Table 3, we present the key findings for the political interest, political talk, and efficacy of Mexican elections models. We also report the effective sample size of the treatment and control groups after weighting in Additional file 1: Tables S1–3. The coefficient, standard error, and *p*-value for the coefficient of the treatment variable and all covariates are reported in the Additional file 1: Tables S1–3.

**Table 3:** Migration effect of immigrant relatives and remittances on political interest, talk, and efficacy of Mexican elections

	Treatment 1: Has immigrant relative in U.S						Treatment 2: Has immigrant relative in U.S & receives remittances					
	Wave 1		Wave 2		Wave 3		Wave 1		Wave 2		Wave 3	
	Coeff.	SE	Coeff.	SE	Coeff.	SE	Coeff.	SE	Coeff.	SE	Coeff.	SE
Interest in Politics	–0.20	0.21	0.22	0.25	0.10	0.25	–0.57	0.30	0.62	0.36	0.94	0.37*
Political Talk	0.55	0.2**	0.13	0.19	0.44	0.2*	–0.18	0.27	0.06	0.27	–0.33	0.27
Efficacy of Elections					–0.12	0.22					–0.77	0.33*

Source: Lawson et al. (2007). Authors' calculations using doubly robust estimation with propensity score weighting in R. Control variables omitted. *p < 0.05 **p < 0.01 ***p < 0.001

Across the three panel waves, having migrant social ties *and* receiving remittances is a positive and significant predictor of political interest (treatment 2). In pre-election wave 1, for those that have only a relative living in the U.S. (treatment 1) 73% have at least some interest in politics, whereas slightly fewer of those without a close immigrant relative are interested in politics at 69%. If those in the treatment group did not have immigrant relatives in the U.S., the percentage would be 78%, reducing the probability of having interest in politics by almost 6%. However, the *p*-value is not significant in any of the panel waves for those respondents who only have migrant social ties.^8^

By contrast, having both a close relative in the U.S. *and* receiving remittances is related to being interested in Mexican politics. In panel waves 1 and 2, respondents that report both forms of migration exposure are more likely to be interested in politics at the 10% level in the pre-election wave and at the 5% level in in the post-election wave 3. The results show a change in the sign of the DR estimator between the first and subsequent waves, suggesting respondents with a close migrant relative that also receive remittances are more likely to become interested in politics over the course of the 2006 election cycle. In wave 3, the unweighted average outcome of respondents reporting interest in politics for the treatment and control groups were 83 and 82%, respectively. This suggests that the treatment group was only slightly larger than the control group. However, after the DR estimation, the weighted average outcome for the treatment group if they had been in the control group decreases to 75%. This tells us that citizens also receiving remittances through the migrant social network increases the probability of being interested in politics by 8%.

Having a relative in the U.S. has a different effect on talking about politics than on political interest. While exposure to international migration operationalized as having close relative (s) in the U.S. (treatment 1) does not affect the probability of being interested in politics in any of the panel waves, it does have a substantive and significant effect on talking about Mexican politics in waves 1 and 3. The implicit and/or explicit political information transmitted through respondents’ transnational social networks positively affects talking about politics with others whereas the addition of receiving remittances through kin and friend networks has no joint impact. Having a close relative in the U.S. increases the probability of talking about politics by 4% in wave 1, and by 5%, in wave 3.^9^ Respondents’ social connections to U.S. family independently impacts political talk, although does not affect their interest in politics unless they also receive remittances. We note, though, the data suggests that in general many fewer respondents report talking about politics compared to having interest in politics; 30% less of the total treatment group sample and 33% less of the total control group sample talked about politics at all compared to levels of political interest.

Finally, in the post-election wave, respondents were asked whether they agree or disagree that Mexican elections are free and fair, giving us some insight into how efficacious Mexican voters believe elections to be. The DR estimates show that the unweighted treatment groups are more likely to agree that Mexican elections are free and fair (77% for treatment 1 and 76% treatment 2) than the unweighted control groups (71 and 74%). However, the DR estimator shows that the weighted average outcome for the treatment group had they been in the control group increases the probability that respondents agree that elections are free and fair for treatment two. Therefore, having a relative in the U.S. and receiving remittances *reduces* the probability that Mexican voters agree that elections are free and fair (8%). In other words, the ideational values and material resources respondents receive through their cross-border social connections to migrants in the U.S. makes them 8% more likely to be critical of Mexican elections. Finally, we note that we estimate a third treatment effect, only receiving remittances abroad, but not from close family members, which captures 20% of the sample. In none of the specifications does only receiving remittances have an effect on our measures of political interest and attitudes.^10^

In addition to reporting the treatment effect estimations, the DR models allow us to assess the relative influence of the covariates estimated and how well the propensity score matching panels are balanced.^11^ First, region, education and income are consistently the top three covariates correlated to both migrant social ties and remittances effects across all models, while the urban dummy variable is more strongly correlated to having both social ties and remittances in addition to the other three variables.^12^ Second, the DR estimations report the weighted mean of each covariate for both the control and treatment groups as well as the Kolmogorov-Smirnov test (K-S test) results.^13^ Results from the K-S test suggest that the treatment and control groups are well balanced across all covariates, with only very small differences between treatment and control. For example, the average K-S result for the DR models in which the treatment effect is significant is 0.0007.

## Discussion

The doubly robust models provide several key insights regarding the role of international migration on political interest, talk, and attitudes about Mexican elections for the non-migrant Mexican electorate. The data reveal that cross-border social networks and monetary resources have independent and joint effects on three outcomes: talking about politics; being interested in politics; and believing that elections are free and fair institutions in Mexico. First, having migrant family ties independently explains the frequency with which individuals talk about politics before and after the presidential election. While we cannot describe the content or precise pathway of the political information migrant family members abroad share with their kin, we do know that that social exposure to international migration, via kinship ties to relatives living in the United States, has a positive effect on non-migrants’ political interest.

To probe the role of social ties on political talk a little deeper, we also test whether the density and type of relationship type has a direct effect on non-migrant political interest. The survey includes a question asking respondents to select the kinds of kinship ties they maintain in the U.S. including spouse, parent (s), children, sibling (s), uncle (s) and/or aunt (s), grandparent (s), cousin (s), niece (s) and/or nephew (s). We created two dichotomous variables from this question. First, we estimate the independent effect of relationship type in a variable that takes the value of one if the social tie was “close” (spouse, child, sibling) and zero if the social tie involved a “distant” relative. Relationship type had no effect on any non-migrant political awareness indicators. Second, we estimate if the density of social ties plays a significant role in the political talk of non-migrants with their political discussion partners. Recall that we estimate the treatment effect of having “any” or “no” migrant relatives in the U.S. in the initial political talk model. DR estimates show that having two or more kinship ties in the U.S. does have a positive and significant effect on political talk in the pre-election and post-election panel waves. Mexican non-migrant voters are 5% more likely to discuss politics if they had more than two migrant social ties in the first wave and 6% more likely in the last wave. Neither the relationship type nor number of ties treatment variables had independent effects on any other dependent variable (political interest or perceived efficacy of elections).^14^

Our results suggest that interpersonal social connections stretching across borders are a necessary, but not sufficient condition of increasing interest in politics or affecting opinions of Mexican elections. Receiving monetary remittances from migrant relatives abroad yields an additional effect that triggers more criticism of Mexican elections and interest in politics, more generally. The acquisition of material resources from migrant kin abroad may reflect tighter social cohesion and in turn, the implicit and explicit political information conveyed through the network may be of greater salience to the non-migrant recipient. While non-migrants are benefitting from international migration by receiving additional household revenue, which helps to mitigate risks even without making the sojourn abroad, stay-at-homes surely bear some of the burden of the migration, whether caring for family or property or keeping businesses afloat in migrants’ absence. Receiving monetary resources from the U.S., which both enable and constrain current and future prospects for migrant households, may strengthen the quality of the social bonds in the transnational social network and thus the political information and opinions shared by individuals becomes more salient to non-migrant voters. Whether the content of the political information is direct or indirect or whether non-migrant recipients agree or disagree is beyond the scope of this paper. The key insight our data reveals is that international migration influences the political interest of Mexican non-migrant through cross-border social networks when they also receive remittances through their network ties. Additionally, non-migrants with more migration exposure become more critical of Mexican elections and interested in politics.

## Conclusion

The political and social logic of international migration produces international families. Migrants and stay-at-homes pursue entwined survival strategies: migrants relocate to a developed society to gain access to the resources that can only be found there, in turn channeling those gains back home in order to stabilize, secure, and improve the options of the kin network remaining in place. However, the migrants are also dependent on the stay at homes, whether providing care to the elderly or to children, looking after property and other investments, and furnishing assistance when problems in the society of destination compel the migrants to look homeward for help. In today’s world, moreover, these decisions to build family economies across borders reflect the additional impact of receiving states’ ever intensifying efforts to police national boundaries. While leaving home for life abroad requires both finances and social capital, those resources no longer suffice; migrants need to find a way through or around control systems. Since not every family member can penetrate borders with equal ease, those most able to cross go first. Consequently, other kin members are left home to wait, remaining there until a visa allows for legal passage or resources permit yet another unauthorized crossing.

While the locus of the migrant’s key connections tends to shift over time, that transition may be highly protracted. Regardless of the motivation leading any one family or individual to leave home, the core familial network almost always moves gradually, erratically, and incompletely. As the migrant has but limited influence over the locational decisions made by the various persons comprising the kin network, some significant other is usually to be found at home. Because other commitments, such as property ownership, further keep emigrants rooted in the place from which they began, inertia exercises considerable weight. These connections may explain why all politics need not necessarily be local, but can, under the circumstances generated by migration, provide both the mechanisms and the motivations – among both migrants and stay-at-homes – for political signals to cross borders.

On the other hand, the possession of ties to some relative abroad may by a necessary, but not sufficient condition for the activation of political activity from afar. As time goes on, increasingly large portions of the home society are connected through social ties to relatives and friends living abroad. In a country like Mexico, with a century-plus long history of migration, roughly one out of two persons living *in* Mexico has a relative living *in* the United States; a trait so commonly shared is unlikely to produce much political variance. Moreover, to exercise influence, those ties have to function as circuits of exchange, providing the vectors whereby information, ideas, and resources move back and forth from place of origin to place of destination (Lacroix et al., 2016). Yet, keeping up those ties requires commitment, which is why migrants maintain cross-border connections in selective fashion. The motivation for doing so may be sapped as the core familial network shifts from country of emigration to country of immigration: the needs associated with life in the high-cost society of residence are likely to reduce the capacity to help out those abroad; over time, distance, separation, and exposure to a different society and way of life makes the immigrants increasingly different from those they left behind. However, while cross-border ties are occasionally cut and often attenuated, in other cases, migrants’ material and affective commitments remain firmly implanted in the country of origin. Under those conditions, migrants and stay-at-homes remain interconnected and cross-border political effects including an increase in the political interest and awareness of stay-at-homes may be more likely in country cases beyond Mexico. As this article shows, those ties provide reasons for migrants residing abroad to pay attention to political matters at home and for relatives remaining at home to attend to the preferences of their migrant kin who have opted for life in a foreign country.

## Additional file

Additional file 1: **Table S1.** Migration effects of immigrant relatives and receiving remittances on interest in politics. **Table S2.** Migration effects of immigrant relative and receiving remittances on political talk. **Table S3.** Migration effects of immigrant relative and receiving remittances on efficacy of Mexican elections. (DOCX 36 kb)

## Footnotes

1 The GBM is a downloadable R package developed by Greg Ridgway that is an algorithm for iteratively forming a collection of simple regression tree models to estimate propensity scores. The GBM’s nonparametric nature reduces the chance of model misspecification.

2 Senior Project Personnel for the Mexico 2006 Panel Study include (in alphabetical order): Andy Baker, Kathleen Bruhn, Roderic Camp, Wayne Cornelius, Jorge Domínguez, Kenneth Greene, Joseph Klesner, Chappell Lawson (Principal Investigator), Beatriz Magaloni, James McCann, Alejandro Moreno, Alejandro Poiré, and David Shirk. Funding for the study was provided by the National Science Foundation (SES-0517971) and Reforma newspaper; fieldwork was conducted by Reforma newspaper’s Polling and Research Team, under the direction of Alejandro Moreno. http://mexicopanelstudy.mit.edu/

3 We note that the survey did not include a question probing for cross-border exchanges other than those involving remittances.

4 Pearson chi-square test = 120.91; *p* = 0.000.

5 Race serves as a proxy for indigenous respondents, which are the poorest and most marginalized groups in Mexico. Previous research shows that race and ethnicity plays a role in shaping political interest, personal efficacy, attitudes and engagement in the electoral process (see Leighley & Vedlitz, 1999).

6 We also evaluate how frequency of following the presidential elections is conditioned by migration exposure in specifications not reported due to space constraints. A question on the survey asks: How closely are you following the presidential campaign: a lot, some, a little, or none? We evaluate this outcome (positive and statistically significant for treatment 1, no effect for treatment 2), but do not report these effects.

7 Note: the question asking respondents whether they agree or disagree that Mexican elections are free and fair only appears in panel wave 3.

8 In the specifications in which we use all categories of response (a lot, some, a little, none) as continuous, we note positive and statistically significant effects for treatment 1 as well. Having a relative in the US increases the likelihood of political interest from one category (“some”) to another (“a lot”) by 20% on average. There is some caution warranted when interpreting ordinal variables as continuous as it may violate some OLS assumptions. However, since the distribution is not skewed, the practical effect is minor and the simplicity of interpreting an OLS should outweigh the technical correctness of ordered logit (see Angrist & Pischke, 2008).

9 The DR results for the political talk models when using the full ordinal values (daily, a few days a week, a few days a month, rarely, never) and interpreting them as continuous values yield similar, albeit stronger results across all three panel waves (from 22 to 24 to 20% across waves 1, 2, and 3, respectively). For example, a respondent with a US social tie (treatment 1) is about 24% more likely, on average, to report talking about politics “a few days a week” than “a few days a month” in wave 3.

10 These results are available by request.

11 We also assess the potential role of media exposure across treatment and control groups using a survey question that asks whether respondents watch news on TV and how frequently. Media exposure across groups was not systematically different and did not affect any of the regression results. We thank reviewer 2 for this suggestion.

12 The covariates relative influence on the treatment effects estimated by GBM are not reported here. We also included an additional contextual variable indicating the percent of international migration living abroad, but the results were not statistically significant and produced no changes in the other covariates.

13 The K-S test compares two samples by quantifying the distance between the empirical distribution functions of two samples, in this case the treatment and control groups.

14 We do not report the results here due to space limitations, but will provide if requested.
