# Supplementary material for: Foreign connections and the difference they make: how migrant ties influence political interest and attitudes in Mexico
Source: Comp Migr Stud. 2018 Nov 19;6(1):35. doi: 10.1186/s40878-018-0098-y (PMC6245234; doi:10.1186/s40878-018-0098-y)
Supplement: Supplementary file 1 — Table S1. Migration effects of immigrant relatives and receiving remittances on interest in politics. Table S2. Migration effects of immigrant relative and receiving remittances on political talk. Table S3. Migration effects of immigrant relative and receiving remittances on efficacy of Mexican elections. (DOCX 36 kb) [file 40878_2018_98_MOESM1_ESM.docx]

| Table S1: Migration Effects of Immigrant Relatives and Receiving Remittances on Interest in Politics | | | | | | | | | | | | | | | | | |  |
| --- | --- | --- | --- | --- | --- | --- | --- | --- | --- | --- | --- | --- | --- | --- | --- | --- | --- | --- |
|  | **Has Immigrant Relative in U.S. (Treatment 1)** | | | | | | | |  | **Has Immigrant Relative in U.S. & Receives Remittances (Treatment 2)** | | | | | | | |  |
|  | Wave 1 | |  | Wave 2 | |  | Wave 3 | |  | Wave 1 | |  | Wave 2 | |  | Wave 3 | |  |
|  | Coeff | SE |  | Coeff | SE |  | Coeff | SE |  | Coeff | SE |  | Coeff | SE |  | Coeff | SE |  |
| **Migration Treatment** | -0.20 | 0.21 |  | 0.22 | 0.25 |  | 0.10 | 0.25 |  | -0.57 | 0.30 | . | 0.62 | 0.36 | . | 0.94 | 0.37 | * |
| **Urban** |  |  |  |  |  |  |  |  |  |  |  |  |  |  |  |  |  |  |
| Rural | 0.44 | 0.25 |  | 0.29 | 0.32 |  | 0.23 | 0.31 |  | 0.28 | 0.40 |  | 0.13 | 0.46 |  | 0.36 | 0.48 |  |
| Mixed | -0.16 | 0.29 |  | -0.18 | 0.36 |  | -0.50 | 0.35 |  | 0.45 | 0.55 |  | -0.45 | 0.66 |  | -0.49 | 0.61 |  |
| **Region** |  |  |  |  |  |  |  |  |  |  |  |  |  |  |  |  |  |  |
| South | -0.52 | 0.28 | * | -0.75 | 0.33 | * | -0.41 | 0.32 |  | -0.52 | 0.41 |  | -0.58 | 0.49 |  | -0.62 | 0.44 |  |
| Center | -0.15 | 0.28 |  | 0.17 | 0.33 |  | -0.16 | 0.33 |  | 0.42 | 0.52 |  | 0.86 | 0.50 | . | -0.05 | 0.63 |  |
| Metro | -0.76 | 0.25 |  | -0.12 | 0.31 |  | -0.27 | 0.29 |  | -1.17 | 0.44 | ** | 0.58 | 0.50 |  | 0.39 | 0.51 |  |
| **Schooling** |  |  |  |  |  |  |  |  |  |  |  |  |  |  |  |  |  |  |
| Incompl. Primary | 0.33 | 0.48 |  | 0.18 | 0.54 |  | 1.30 | 0.48 | ** | -0.10 | 0.80 |  | 0.71 | 0.68 |  | 1.66 | 0.63 | ** |
| Complete Primary | 1.14 | 0.49 |  | 0.68 | 0.56 |  | 1.26 | 0.48 | ** | 1.58 | 0.88 | . | 1.46 | 0.70 | * | 1.52 | 0.70 | * |
| Incompl. Middle | 0.99 | 0.63 |  | -0.22 | 0.70 |  | 1.02 | 0.66 |  | -1.41 | 1.31 |  | -1.49 | 1.03 |  | -0.58 | 1.23 |  |
| Complete Middle | 1.39 | 0.52 |  | 0.21 | 0.60 |  | 1.81 | 0.55 | *** | 0.90 | 0.90 |  | 0.42 | 0.76 |  | 2.30 | 0.82 | ** |
| Incompl. High | 1.90 | 0.66 | . | 1.32 | 0.78 |  | 2.96 | 0.81 | *** | 0.97 | 1.05 |  | 2.17 | 1.03 | * | 4.91 | 1.29 | *** |
| High School/GED | 1.39 | 0.56 |  | 0.33 | 0.65 |  | 2.46 | 0.62 | *** | 0.56 | 1.00 |  | -0.25 | 0.97 |  | 2.86 | 1.07 | ** |
| Incompl. College | 1.90 | 0.67 | . | 2.21 | 1.17 |  | 3.32 | 0.96 | *** | 2.47 | 1.15 | * | 2.73 | 1.28 | * | 4.39 | 1.44 | ** |
| College or more | 2.71 | 0.72 | * | 1.65 | 0.81 | * | 3.47 | 0.83 | *** | 0.41 | 1.22 |  | 0.92 | 0.93 |  | 3.10 | 1.12 | ** |
| **Income** |  |  |  |  |  |  |  |  |  |  |  |  |  |  |  |  |  |  |
| 1,300 - 1,999 | -0.02 | 0.35 | * | 0.92 | 0.47 | * | 1.00 | 0.42 | * | -0.49 | 0.52 |  | -0.19 | 0.54 |  | 0.22 | 0.56 |  |
| 2,000 - 2,599 | -0.03 | 0.38 |  | -0.16 | 0.44 |  | 0.31 | 0.42 |  | -0.02 | 0.58 |  | -0.84 | 0.63 |  | -0.47 | 0.55 |  |
| 2,600 - 3,999 | -0.49 | 0.34 |  | 0.02 | 0.40 |  | 0.30 | 0.39 |  | -1.09 | 0.60 | . | -0.17 | 0.54 |  | -0.06 | 0.64 |  |
| 4,000 - 5,199 | -0.21 | 0.36 |  | 0.06 | 0.44 |  | 0.47 | 0.42 |  | -0.49 | 0.60 |  | -0.15 | 0.81 |  | 1.53 | 0.84 | . |
| 5,200 - 6,499 | -0.38 | 0.42 |  | 0.14 | 0.51 |  | 0.75 | 0.48 |  | 0.07 | 0.73 |  | -0.01 | 0.63 |  | 0.44 | 0.70 |  |
| 6,500 - 7,899 | -0.18 | 0.55 |  | 0.47 | 0.66 |  | 0.83 | 0.81 |  | 0.77 | 0.87 |  | 1.65 | 0.93 | . | 1.87 | 0.92 | * |
| 7,900 - 9,199 | 0.19 | 0.53 |  | 0.34 | 0.67 |  | 0.54 | 0.60 |  | -0.11 | 0.86 |  | 2.11 | 1.01 | * | 2.23 | 0.89 | * |
| 9,200 - 10,499 | 0.75 | 0.70 | . | 1.78 | 1.03 |  | 0.24 | 0.78 |  | 0.26 | 0.79 |  | 0.66 | 1.05 |  | -0.20 | 0.84 |  |
| 10,500 or more | 0.11 | 0.50 | * | 1.76 | 0.84 | * | 0.62 | 0.60 |  | 1.21 | 0.84 |  | 3.22 | 1.03 | ** | 2.05 | 1.08 | . |
| **Age** |  |  |  |  |  |  |  |  |  |  |  |  |  |  |  |  |  |  |
| 31 - 46 | 0.54 | 0.27 |  | -0.29 | 0.33 |  | 0.03 | 0.30 |  | 0.02 | 0.41 |  | -1.34 | 0.64 | * | 0.09 | 0.43 |  |
| 46 or older | 0.48 | 0.31 | * | -0.88 | 0.35 | * | 0.05 | 0.35 |  | -0.49 | 0.48 |  | -2.23 | 0.64 | *** | 0.16 | 0.52 |  |
| **Gender** | 0.04 | 0.20 |  | 0.29 | 0.26 |  | 0.57 | 0.24 | * | -0.30 | 0.30 |  | 0.14 | 0.33 |  | -0.05 | 0.37 |  |
| **Marital Status** |  |  |  |  |  |  |  |  |  |  |  |  |  |  |  |  |  |  |
| Married | -0.38 | 0.28 |  | 0.05 | 0.33 |  | 0.13 | 0.31 |  | 0.12 | 0.43 |  | 0.88 | 0.60 |  | 1.03 | 0.42 | * |
| Other | 0.09 | 0.34 |  | 0.21 | 0.43 |  | 0.70 | 0.39 | . | 0.34 | 0.54 |  | 0.06 | 0.79 |  | 1.41 | 0.49 | ** |
| **Church Attendance** | |  |  |  |  |  |  |  |  |  |  |  |  |  |  |  |  |  |
| Monthly | 0.15 | 0.29 |  | -0.15 | 0.35 |  | 0.03 | 0.34 |  | -0.08 | 0.47 |  | -0.57 | 0.51 |  | 0.30 | 0.64 |  |
| Special Occasion | -0.45 | 0.22 |  | -0.28 | 0.29 |  | -0.23 | 0.27 |  | 0.24 | 0.36 |  | -0.38 | 0.41 |  | -0.16 | 0.40 |  |
| Never | -1.21 | 0.43 | * | -1.03 | 0.49 | * | -0.96 | 0.52 | . | 0.33 | 0.74 |  | 0.11 | 0.93 |  | 0.03 | 0.69 |  |
| Don't know/Refused | -2.32 | 0.72 |  | -0.23 | 0.89 |  | 0.18 | 1.02 |  | 0.19 | 0.99 |  | 0.85 | 1.28 |  | 0.56 | 1.03 |  |
| **Race** |  |  |  |  |  |  |  |  |  |  |  |  |  |  |  |  |  |  |
| Light brown | 0.29 | 0.25 |  | 0.19 | 0.32 |  | 0.11 | 0.29 |  | 0.53 | 0.47 |  | -1.08 | 0.54 | * | 0.03 | 0.44 |  |
| Dark Brown | -0.15 | 0.27 |  | -0.10 | 0.36 |  | -0.04 | 0.32 |  | 0.28 | 0.52 |  | -1.30 | 0.63 | * | -0.02 | 0.48 |  |
| Other | -4.61 | 1.56 | *** | 12.67 | 0.98 | *** | -4.67 | 1.59 | ** | - | - |  | - | - |  | - | - |  |
| No Answer | -14.45 | 0.83 | *** | -14.86 | 1.16 | *** | -4.36 | 1.34 | ** | - | - |  | - | - |  | - | - |  |
| **Intercept** | 0.46 | 0.62 |  | 1.29 | 0.80 |  | -0.67 | 0.72 |  | 0.92 | 0.91 |  | 2.87 | 1.06 | ** | -1.71 | 1.03 | . |
| Source: Lawson et al. 2007. Authors' calculations using doubly robust estimation with propensity score weighting in R. Omitted categories: north, no schooling, 0-1299, <31 years old, female, single, attend weekly or more, white. Signif. codes: . p < 0.10 *p < 0.05 **p < 0.01 ***p < 0.001 | | | | | | | | | | | | | | | | | | |

| Table S2: Migration Effects of Immigrant Relative and Receiving Remittances on Political Talk | | | | | | | | | | | | | | | | | |  |
| --- | --- | --- | --- | --- | --- | --- | --- | --- | --- | --- | --- | --- | --- | --- | --- | --- | --- | --- |
|  | **Has Immigrant Relative in U.S. (Treatment 1)** | | | | | | | |  | **Has Immigrant Relative in U.S. & Receives Remittances (Treatment 2)** | | | | | | | |  |
|  | Wave 1 | |  | Wave 2 | |  | Wave 3 | |  | Wave 1 | |  | Wave 2 | |  | Wave 3 | |  |
|  | Coeff | SE |  | Coeff | SE |  | Coeff | SE |  | Coeff | SE |  | Coeff | SE |  | Coeff | SE |  |
|  |  |  |  |  |  |  |  |  |  |  |  |  |  |  |  |  |  |  |
| **Migration Exposure** | 0.55 | 0.20 | ** | 0.13 | 0.19 |  | 0.44 | 0.20 | * | -0.18 | 0.27 |  | 0.06 | 0.27 |  | -0.33 | 0.27 |  |
| **Urban** |  |  |  |  |  |  |  |  |  |  |  |  |  |  |  |  |  |  |
| Rural | -0.05 | 0.27 |  | -0.08 | 0.26 |  | -0.17 | 0.27 |  | 0.32 | 0.33 |  | -0.04 | 0.34 |  | 0.95 | 0.37 | ** |
| Mixed | 0.52 | 0.26 | * | 0.40 | 0.25 |  | 0.74 | 0.24 | ** | -1.01 | 0.39 | ** | -1.79 | 0.45 | *** | -0.31 | 0.46 |  |
| **Region** |  |  |  |  |  |  |  |  |  |  |  |  |  |  |  |  |  |  |
| South | 0.06 | 0.25 |  | 0.06 | 0.23 |  | 0.41 | 0.23 | . | 0.20 | 0.40 |  | 0.06 | 0.36 |  | 0.06 | 0.36 |  |
| Center | -0.03 | 0.24 |  | 0.37 | 0.25 |  | 0.28 | 0.25 |  | 0.35 | 0.46 |  | 0.82 | 0.52 |  | 1.42 | 0.44 | ** |
| Metro | -0.41 | 0.28 |  | -0.20 | 0.27 |  | 0.03 | 0.26 |  | -0.32 | 0.38 |  | 0.33 | 0.38 |  | 0.57 | 0.37 |  |
| **Schooling** |  |  |  |  |  |  |  |  |  |  |  |  |  |  |  |  |  |  |
| Incompl. Primary | 0.42 | 0.55 |  | 0.40 | 0.57 |  | 0.98 | 0.55 | . | -0.75 | 0.68 |  | 1.01 | 0.65 |  | -0.44 | 0.70 |  |
| Compl. Primary | 1.51 | 0.53 | ** | 0.85 | 0.56 |  | 1.10 | 0.53 | * | 1.39 | 0.68 | * | 1.45 | 0.62 | * | 0.59 | 0.62 |  |
| Incompl. Middle | 1.48 | 0.65 | * | 0.95 | 0.65 |  | 1.14 | 0.62 | . | 1.06 | 0.86 |  | 0.65 | 0.83 |  | -0.21 | 0.84 |  |
| Compl. Middle | 1.28 | 0.56 | * | 0.80 | 0.59 |  | 1.60 | 0.55 | ** | 0.90 | 0.76 |  | 1.39 | 0.70 | * | 0.92 | 0.71 |  |
| Incompl. HS | 2.70 | 0.61 | *** | 1.82 | 0.63 | ** | 2.50 | 0.62 | *** | 2.55 | 0.95 | ** | 1.97 | 0.89 | * | 2.26 | 0.87 | ** |
| HS/GED | 2.47 | 0.58 | *** | 1.65 | 0.61 | ** | 2.04 | 0.57 | *** | 2.06 | 0.75 | ** | 2.45 | 0.77 | ** | 1.07 | 0.73 |  |
| Incompl. College | 2.23 | 0.64 | *** | 1.79 | 0.72 | * | 1.87 | 0.63 | ** | 2.31 | 0.86 | ** | 0.23 | 0.96 |  | 0.49 | 0.94 |  |
| College or more | 3.26 | 0.62 | *** | 2.16 | 0.65 | *** | 3.10 | 0.62 | *** | 1.45 | 0.99 |  | 1.53 | 0.92 | . | 1.72 | 0.81 | * |
| **Income** |  |  |  |  |  |  |  |  |  |  |  |  |  |  |  |  |  |  |
| 1,300 - 1,999 | 0.39 | 0.24 |  | 0.12 | 0.24 |  | 0.17 | 0.22 |  | -0.25 | 0.53 |  | -0.64 | 0.49 |  | 0.18 | 0.47 |  |
| 2,000 - 2,599 | 0.78 | 0.29 | ** | 0.39 | 0.29 |  | 0.10 | 0.26 |  | -0.22 | 0.60 |  | -0.73 | 0.51 |  | 0.66 | 0.55 |  |
| 2,600 - 3,999 | -0.54 | 0.38 |  | -0.15 | 0.38 |  | 0.34 | 0.39 |  | -0.39 | 0.48 |  | -0.09 | 0.50 |  | 0.57 | 0.46 |  |
| 4,000 - 5,199 | -0.18 | 0.40 |  | -0.05 | 0.36 |  | 0.71 | 0.39 | . | 0.29 | 0.58 |  | -0.26 | 0.51 |  | 0.58 | 0.56 |  |
| 5,200 - 6,499 | -0.35 | 0.37 |  | 0.04 | 0.35 |  | 0.42 | 0.36 |  | -0.60 | 0.60 |  | -0.19 | 0.55 |  | 1.06 | 0.58 | . |
| 6,500 - 7,899 | -0.21 | 0.38 |  | 0.24 | 0.36 |  | 0.94 | 0.38 | * | 0.76 | 0.73 |  | 1.19 | 0.63 | . | 2.06 | 0.64 | ** |
| 7,900 - 9,199 | -0.45 | 0.40 |  | 0.07 | 0.39 |  | 1.06 | 0.41 | * | 0.46 | 0.69 |  | 0.70 | 0.77 |  | 0.38 | 1.02 |  |
| 9,200 - 10,499 | -0.07 | 0.46 |  | 0.65 | 0.51 |  | 0.81 | 0.46 | . | 0.57 | 0.67 |  | 0.34 | 0.62 |  | 1.57 | 0.68 | * |
| 10,500 or more | -0.51 | 0.49 |  | 0.37 | 0.46 |  | -0.02 | 0.47 |  | 0.57 | 0.89 |  | 2.51 | 0.69 | *** | 0.88 | 0.56 |  |
| **Age** |  |  |  |  |  |  |  |  |  |  |  |  |  |  |  |  |  |  |
| 31 - 46 | 0.17 | 0.50 |  | 0.67 | 0.56 |  | 0.93 | 0.56 | . | -0.10 | 0.40 |  | -0.70 | 0.35 | * | -0.64 | 0.37 | . |
| 46 or older | 0.32 | 0.45 |  | 1.80 | 0.53 | *** | 0.79 | 0.45 | . | 0.77 | 0.43 | . | -0.50 | 0.43 |  | -0.86 | 0.43 | * |
| **Gender** | 0.31 | 0.19 |  | 0.39 | 0.19 | * | 0.27 | 0.18 |  | 0.50 | 0.30 | . | 0.33 | 0.28 |  | 0.71 | 0.29 | * |
| **Marital Status** |  |  |  |  |  |  |  |  |  |  |  |  |  |  |  |  |  |  |
| Married | -0.07 | 0.25 |  | -0.11 | 0.25 |  | 0.15 | 0.24 |  | 0.18 | 0.37 |  | 0.55 | 0.35 |  | 0.57 | 0.41 |  |
| Other | 0.14 | 0.31 |  | -0.03 | 0.31 |  | 0.39 | 0.28 |  | -0.30 | 0.49 |  | 0.36 | 0.44 |  | 0.61 | 0.46 |  |
| **Church Attendance** | |  |  |  |  |  |  |  |  |  |  |  |  |  |  |  |  |  |
| Monthly | -0.09 | 0.10 |  | -0.07 | 0.10 |  | -0.03 | 0.09 |  | -0.10 | 0.16 |  | -0.13 | 0.14 |  | -0.20 | 0.14 |  |
| Special Occasion | - | - |  | - | - |  | - | - |  | - | - |  | - | - |  | - | - |  |
| Never | - | - |  | - | - |  | - | - |  | - | - |  | - | - |  | - | - |  |
| Don't know/Refused | - | - |  | - | - |  | - | - |  | - | - |  | - | - |  | - | - |  |
| **Race** |  |  |  |  |  |  |  |  |  |  |  |  |  |  |  |  |  |  |
| Light brown | -0.40 | 0.24 | . | 0.21 | 0.26 |  | 0.23 | 0.22 |  | -0.29 | 0.37 |  | 0.13 | 0.40 |  | 0.08 | 0.43 |  |
| Dark Brown | -0.69 | 0.26 | ** | 0.02 | 0.28 |  | -0.04 | 0.26 |  | -0.72 | 0.41 | . | 0.15 | 0.45 |  | -0.59 | 0.46 |  |
| Other | 2.74 | 1.45 | . | 3.20 | 1.47 | * | -2.61 | 1.45 | . | - | - |  | - | - |  | - | - |  |
| No Answer | -12.83 | 0.80 | *** | -12.84 | 1.11 | *** | -1.64 | 1.20 |  | - | - |  | - | - |  | - | - |  |
| **Intercept** | -2.18 | 0.68 | ** | -1.59 | 0.73 | * | -3.26 | 0.69 | *** | -1.69 | 0.93 | . | -1.37 | 0.94 |  | -1.82 | 0.92 | * |
| Source: Lawson et al. 2007. Authors' calculations using doubly robust estimation with propensity score weighting in R. Omitted categories: north, no schooling, 0-1299, <31 years old, female, single, attend weekly or more, white. Signif. codes: . p < 0.10 *p < 0.05 **p < 0.01 ***p < 0.001 | | | | | | | | | | | | | | | | | | |

| Table S3: Migration Effects of Immigrant Relative and Receiving Remittances on Efficacy of Mexican Elections | | | | | | |
| --- | --- | --- | --- | --- | --- | --- |
|  | **Has Immigrant Relative in U.S.** | | | **Has Immigrant Relative in U.S. & Receives Remittances** | | |
|  | Coeff. | SE |  | Coeff. | SE |  |
|  |  |  |  |  |  |  |
| **Migration Exposure** | -0.12 | 0.22 |  | -0.77 | 0.33 | * |
| **Urban** |  |  |  |  |  |  |
| Rural | 0.40 | 0.32 |  | -0.53 | 0.40 |  |
| Mixed | 0.06 | 0.31 |  | -0.28 | 0.66 |  |
| **Region** |  |  |  |  |  |  |
| South | 0.16 | 0.35 |  | 0.52 | 0.58 |  |
| Center | -1.42 | 0.30 | *** | -1.33 | 0.63 | * |
| Metro | -0.84 | 0.28 | ** | -1.32 | 0.45 | ** |
| **Schooling** |  |  |  |  |  |  |
| Incompl. Primary | -0.92 | 0.77 |  | -2.82 | 0.82 | *** |
| Complete Primary | -0.71 | 0.75 |  | -2.56 | 0.80 | ** |
| Incompl. Middle | -0.93 | 0.84 |  | -1.52 | 1.40 |  |
| Complete Middle | -1.03 | 0.78 |  | -3.60 | 0.98 | *** |
| Incompl. High | -0.20 | 0.85 |  | -2.02 | 1.19 | . |
| High School/GED | -0.99 | 0.79 |  | -4.46 | 0.99 | *** |
| Incompl. College | -1.08 | 0.85 |  | -3.74 | 1.17 | ** |
| College or more | -0.82 | 0.80 |  | -4.14 | 1.15 | *** |
| **Income** |  |  |  |  |  |  |
| 1,300 - 1,999 | 0.08 | 0.49 |  | 0.06 | 0.64 |  |
| 2,000 - 2,599 | -0.44 | 0.42 |  | -1.51 | 0.66 | * |
| 2,600 - 3,999 | -0.18 | 0.40 |  | -0.47 | 0.62 |  |
| 4,000 - 5,199 | -0.37 | 0.41 |  | -0.09 | 0.63 |  |
| 5,200 - 6,499 | -0.26 | 0.44 |  | -0.54 | 0.69 |  |
| 6,500 - 7,899 | -0.09 | 0.49 |  | -0.92 | 0.79 |  |
| 7,900 - 9,199 | -0.04 | 0.52 |  | 1.49 | 0.76 | . |
| 9,200 - 10,499 | 0.85 | 0.63 |  | 0.72 | 0.91 |  |
| 10,500 or more | 0.38 | 0.51 |  | 0.50 | 1.02 |  |
| **Age** |  |  |  |  |  |  |
| 31 - 46 | -0.04 | 0.26 |  | -0.19 | 0.46 |  |
| 46 or older | -0.25 | 0.32 |  | -0.94 | 0.58 |  |
| **Gender** | 0.03 | 0.21 |  | -0.53 | 0.35 |  |
| **Marital Status** |  |  |  |  |  |  |
| Married | 0.09 | 0.26 |  | -0.11 | 0.43 |  |
| Other | 0.11 | 0.33 |  | -1.29 | 0.54 | * |
| **Church Attendance** |  |  |  |  |  |  |
| Monthly | 0.31 | 0.30 |  | 0.11 | 0.47 |  |
| Special Occasion | -0.20 | 0.23 |  | -0.32 | 0.37 |  |
| Never | 0.04 | 0.49 |  | 0.80 | 1.33 |  |
| Don't know/Refused | -0.46 | 0.75 |  | 0.99 | 1.37 |  |
| **Race** |  |  |  |  |  |  |
| Light brown | -0.32 | 0.27 |  | -1.40 | 0.55 | * |
| Dark Brown | -0.41 | 0.30 |  | -1.23 | 0.55 | * |
| Other | -16.57 | 1.16 | *** | - | - |  |
| No Answer | 12.73 | 1.04 | *** | - | - |  |
| **Intercept** | 3.13 | 0.96 | ** | 8.24 | 1.32 | *** |
| Source: Lawson et al. 2007. Authors' calculations using doubly robust estimation with propensity score weighting in R. Omitted categories: north, no schooling, 0-1299, <31 years old, female, single, attend weekly or more, white. Signif. codes: . p < 0.10 *p < 0.05 **p < 0.01 ***p < 0.001 | | | | | | |
